# Using Fatigue Characteristics to Analyse Test Results for 16Mo3 Steel under Tension-Compression and Oscillatory Bending Conditions

**DOI:** 10.3390/ma13051197

**Published:** 2020-03-06

**Authors:** Andrzej Kurek

**Affiliations:** Faculty of Mechanical Engineering, Opole University of Technology, Mikolajczyka 5 St., 45-271 Opole, Poland; a.kurek@po.opole.pl; Tel.: +48-666-053-692

**Keywords:** tension-compression, oscillatory bending, fatigue

## Abstract

In this study, 16Mo3 steel was analysed for fatigue tests under tension-compression and oscillatory bending conditions. The analysis involved a comparison of fatigue test results obtained using the Manson-Coffin-Basquin, Langer and Kandil models and the models proposed by Kurek-Łagoda. It was observed that it is possible to substitute the basic tension-compression test performed in large testing machines with oscillatory bending tests carried out on a simple, modern test stand. The tests were performed under oscillatory bending on a prototype machine. The testing of 16Mo3 steel proved that the best-known Mason-Coffin-Basquin fatigue characteristic describes the results of all of the experimental tests very well, but the model can only be used when it is possible to divide strains into elastic and plastic components. It should be emphasised here that there is no such possibility in the case of tests performed under oscillatory bending conditions. It was proven that the proposed test method can substitute the tension-compression test very well and be a much more cost efficient way to obtain LCF material fatigue properties.

## 1. Introduction

The process of a structural material’s fatigue is an important topic in contemporary materials management. Numerous studies and modern technologies have provided new possibilities with regards to the use of materials. Notably, 16Mo3 is an EN10028 specified pressure vessel grade chrome molybdenum steel alloy for use in elevated working temperatures. The material is used as a weldable steel in the fabrication of industrial boilers and steel pressurised vessels found in the oil, gas and chemical industry. Due to the materials chrome and molybdenum content, 16Mo3 has excellent heat resistance and corrosion resistant characteristics. The 16Mo3 steel is a structural material that is widely used, particularly in power engineering; therefore, it is the subject of the analysis described in this paper. A correct analysis of this steel in terms of strain and stress is important in both power engineering and the machine-building industry [1]. Fatigue is the main reason for the premature destruction of structures [2,3].

The purpose of this work is to compare the test results for 16Mo3 steel using fatigue characteristics under tension-compression and oscillatory bending conditions according to different models.

In the cases of oscillatory bending and tension-compression, we obtain normal stresses as test results. However, it turns out that the fatigue strength characteristics for oscillatory bending and tension-compression are not the same. To date, only a few authors have attempted to address this issue. In 1996, a comparison of bending and tension-compression was performed by Troshenko, who presented an analysis of stress and strain amplitudes under both bending and tension-compression conditions [4]. A similar analysis was carried out by Manson and Murahidharvan. The results of their work are discussed in [5]. This same issue was also raised in [6] and [7]. An analogous problem was identified by Hassan and Liu [8]. They compared fatigue strengths using different methods of cyclic bending (rotational, four-point, and bending with restrain). They obtained different fatigue life values despite the fact that the bending values corresponded to normal stress each time. As a consequence, the resulting fatigue characteristics differed as well [9,10,11].

However, it is important to carry out all analyses in terms of stress amplitude, strain amplitude and energy. Therefore, all of these aspects were compared in this study. The results of tension-compression tests were taken from [12]. The test results for oscillatory bending were obtained on a test stand that allows for the performance of a test at a controlled strain amplitude.

## 2. Fatigue Characteristics

The Wohler (Basquin) model is a basic stress characteristic that shows the relation between stress amplitude and number of cycles. This relation is expressed in a double-logarithmic system [13]:

(1) $$logN_{f}\;=\;A+m\;log\sigma_{a},$$

where *N_f_* is the fatigue life in cycles, $\sigma_{a}$ is the stress amplitude for tension-compression or bending and *A* and *m* are regression model constants. Equation (1) can be approximated using the following formula:

(2) $$\sigma_{a}\;=\;\;\sigma_{f}^{\prime }{\left(2N_{f}\right)}^{b}.$$

While analysing strain characteristics, the Manson-Coffin-Basquin model and the Langer model should be mentioned. The authors of these strain characteristics proposed an empirical model that makes the amplitude *ε_a,t_* dependent on the number of cycles.

The Manson-Coffin-Basquin (MCB) model is defined as follows:

(3) $$\varepsilon_{a,t}\;=\;\varepsilon_{a,e}+\varepsilon_{a,p}\;=\;\frac{\sigma \prime_{f}}{E}{\left(2N_{f}\right)}^{b}+\varepsilon \prime_{f}{\left(2N_{f}\right)}^{c}$$

where *ε_a,t_* is the total strain amplitude, which is given as the sum of the amplitudes of the elastic strain *ε_a,e_* and the plastic strain *ε_a,p_*; 2*N_f_* is the number half-cycles; *E* is Young’s modulus; *σ′_f_* and *b* are the fatigue life coefficient and exponent, respectively; and *ε′_f_* and *c* are the coefficient and exponent of the fatigue plastic strain, respectively.

In the case where it is possible to determine both the elastic $\varepsilon_{ae}$ and plastic $\varepsilon_{ap}$ components of the total strain $\varepsilon_{at}$, it is recommended that the MCB model (3) be used.

However, when we have to deal with bending, where it is not possible to separate the elastic and plastic components, the MCB model (3) cannot be used. In these cases, the models (4) and (5) are used.

The Langer formula [14] is defined as follows:

(4) $$logN_{f}\;=\;A-B\;log\left(\varepsilon_{a,t}-C\right)$$

where *A, B* and *C* are material constants.

Another formula that is used in fatigue calculations regarding strain is the Kandil model [15]:

(5) $$log\varepsilon_{at}\;=\;A-B\;log\left(N_{f}\right)+C\;{log}^{2}\left(N_{f}\right)$$

where *A, B* and *C* are material constants.

Studies refer to the model (5) very often.

The authors of this article propose the following combination of models (4) and (5):

(6) $$log{\left(\varepsilon_{a,t}-D\right)}_{t}\;=\;A-B\;log\left(N_{f}\right)+C\;{log}^{2}\left(N_{f}\right)$$

where *A, B, C* and *D* are material constants.

It is necessary to use the Ramberg-Osgood Equation (7), which describes the relation between stress amplitude and strain amplitude:

(7) $$\varepsilon_{a,t}\;=\;\varepsilon_{a,e}+\varepsilon_{a,p}\;=\;\frac{\sigma_{a}}{E}+{\left(\frac{\sigma_{a}}{K\prime }\right)}^{\frac{1}{n\prime }}$$

where *σ_a_* is the stress amplitude, *K’* is the cyclic strength coefficient, *n’* is the cyclic strengthening exponent, $\varepsilon_{a,t}$ is the strain amplitude, $\varepsilon_{a,e}$ is the elastic strain amplitude and $\varepsilon_{a,p}$ is the plastic strain amplitude.

Recently, a fatigue characteristic for energy has been used in calculations and during the analysis of test results. The formula for this parameter has the following form [11]:

(8) $$W_{a}\;=\;\frac{\sigma_{a}\varepsilon_{a}}{2}$$

where $W_{a}$ is the stress and strain amplitude of the energy parameter.

For the elastic model $\sigma_{a}$ = $\sigma_{e}$ and the elasto-plastic model $\sigma_{a}$ = $\sigma_{ep}$, we can derive, respectively:

(9) $$W_{e}\;=\;\frac{\sigma_{e}{}^{2}}{2E}$$

and

(10) $$W_{ep}\;=\;\frac{\sigma_{ep}\varepsilon_{ep}}{2}$$

where $W_{e}$ is the amplitude of the energy parameter according to the elastic model and $W_{ep}$ is the amplitude of the energy parameter according to the elasto-plastic model.

The MCB model (3) was developed for tension-compression on the basis of the relation between strain and the number of cycles required for destruction. However, this model is used only when it is possible to separate the elastic and plastic components. A fatigue characteristic for energy has been developed for the MCB model. Substituting the Equations (2) and (3) into (10), we obtain the following:

(11) $$W_{ep}\;=\;\frac{\sigma^{2}{}_{f}}{2E}{\left(2N_{f}\right)}^{2b}+\frac{{\sigma^{\prime }}_{f}{\varepsilon^{\prime }}_{f}}{2}{\left(2N_{f}\right)}^{\left(b+c\right)}{}_{}.$$

In the case of using Equations (2) and (9), we obtain for the elastic component:

(12) $$W_{e}\;=\;\frac{\sigma \prime_{f}{}^{2}}{2E}{\left(2N_{f}\right)}^{2b}.$$

## 3. Fatigue Testing of 16Mo3 Steel under Tension-compression and Oscillatory Bending Conditions

**Figure 1:**
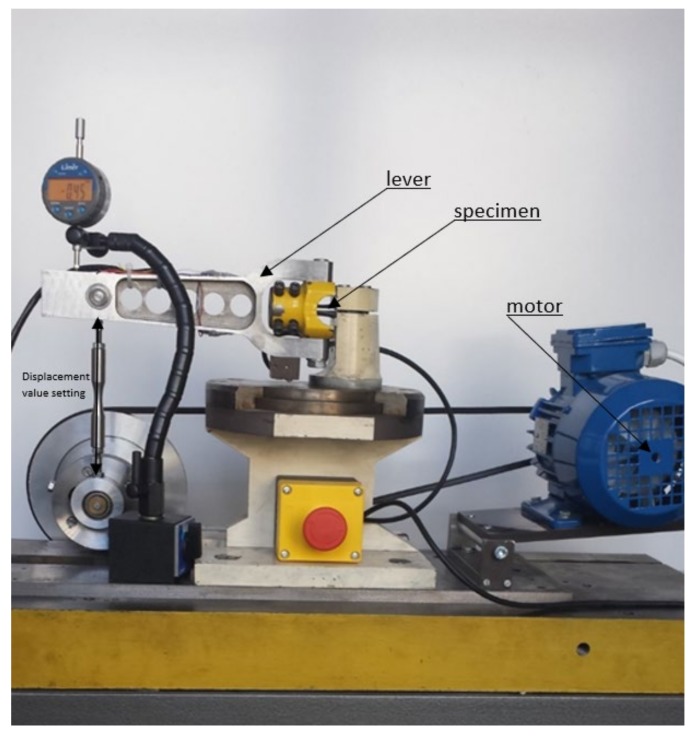
The test stand used to test 16Mo3 steel under oscillatory bending conditions.

The 16Mo3 steel was analysed in terms of tension-compression, the results for which were taken from [16,17] and oscillatory bending. In the case of oscillatory bending, tests were performed using the testing machine in the laboratory at the Opole University of Technology (Figure 1). This machine allows for the control of arm outreach, which makes it possible to obtain a constant strain amplitude and carry out the test and analyse the steel with a controlled strain. 

**Figure 2:**
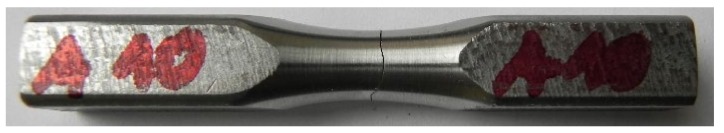
The geometry of a test piece used to test 16Mo3 steel under oscillatory bending conditions.

The test stand (Figure 1) is a prototype test stand. “Diabolo” type samples were used in testing, the shape of which are presented in Figure 2. The tests in this case were carried out at the controlled lever inclination which is reflected (after rescaling) directly in the controlled amplitude of the total strain (ε_at_). A bending moment amplitude was also registered in the course of testing. The machine for strain controlled oscillatory bending is quite simple in its design. A motor controlled with the inverter allows the leaver to bend the specimen. The level of displacement of the lever is set before the rest using roman screw mechanism (as displacement value setting on Figure 1), and the value of bending moment is calculated on the basis of elastic strain deformation of the lever measured using a strain gauge. 

The test pieces that were subjected to bending had geometry characterised by a varying cross-section (of the ‘diabolo’ type) (Figure 2). The tests were carried out under 5 Hz frequency. The big advantage of cyclic bending is that due to the stress and strain gradient [18] in the cross section of the specimen, the amount of heat generated by the testing process is much lower, and most of it occurs on the surface of specimen. This allows the user to conduct a test under much higher frequencies. 

Testing under oscillatory bending conditions allowed us to determine both fatigue life at the initiation point and total fatigue life. At the moment of a crack’s initiation, there was a sudden drop in the bending moment that acted on the test piece, which was correlated with the occurrence of visible cracks in the test piece of approximately 1 mm.

**Figure 3:**
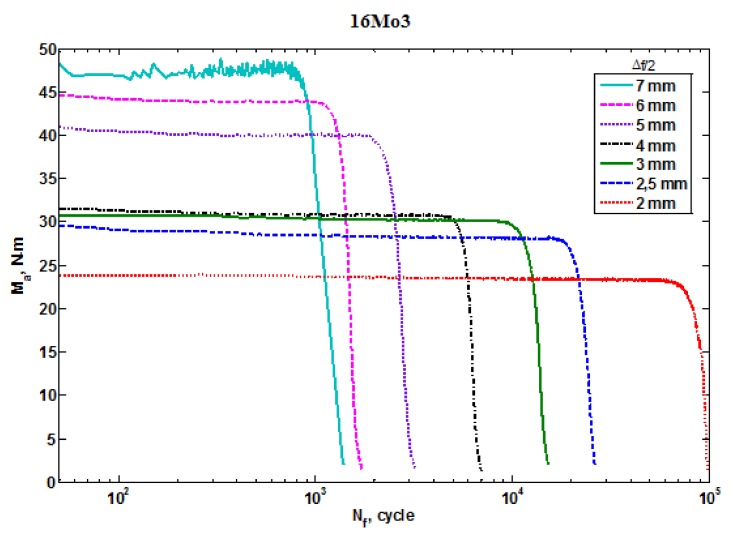
The change in the moments that induce bending, depending on the number of cycles at constant deviation amplitudes.

Figure 3 shows the moment of a crack’s initiation due to fatigue, when the bending moment began to drop rapidly. An analysis of changes in the bending moment showed that greater bending moment amplitudes were obtained in the case of larger deviation amplitudes; thus, the fatigue life values were smaller [19].

**Figure 4:**
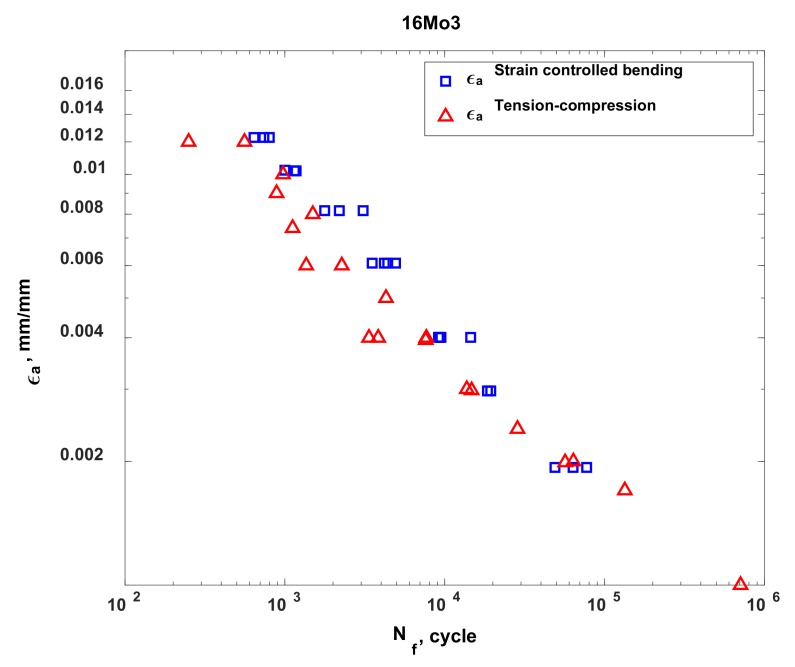
Dependence of strain amplitudes on the number of cycles.

This specific number of cycles was taken to be the number of cycles required to initiate a crack due to fatigue. Figure 4 shows the dependence of strain amplitudes on the number of cycles required to initiate a crack in 16Mo3 steel.

**Figure 5:**
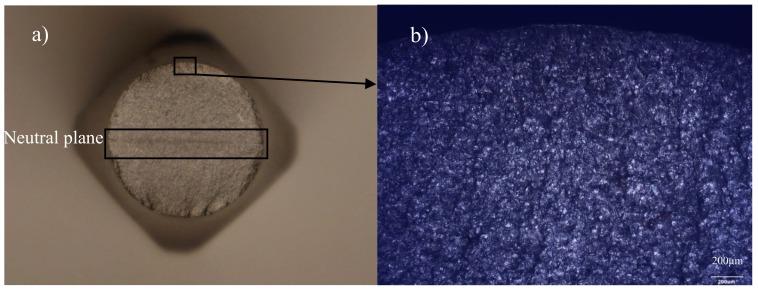
A cross-section with a visible neutral plane (**a**) and microscopic fracture (**b**) of the test piece after fatigue tests under oscillatory bending conditions.

Figure 5a shows a cross-section of the test piece after fatigue tests under oscillatory bending conditions. The figure clearly shows a neutral plane, in relation to which bending has occurred, and the symmetry of the cracking process. Figure 5b shows a microscopic fracture in the test piece after fatigue tests under oscillatory bending conditions.

A comparison of the material constants (Table 1) that appear in Equations (1), (3) and (11) was performed for fatigue tests under tension-compression and oscillatory bending conditions according to the ASTM standard [20].

**Figure 6:**
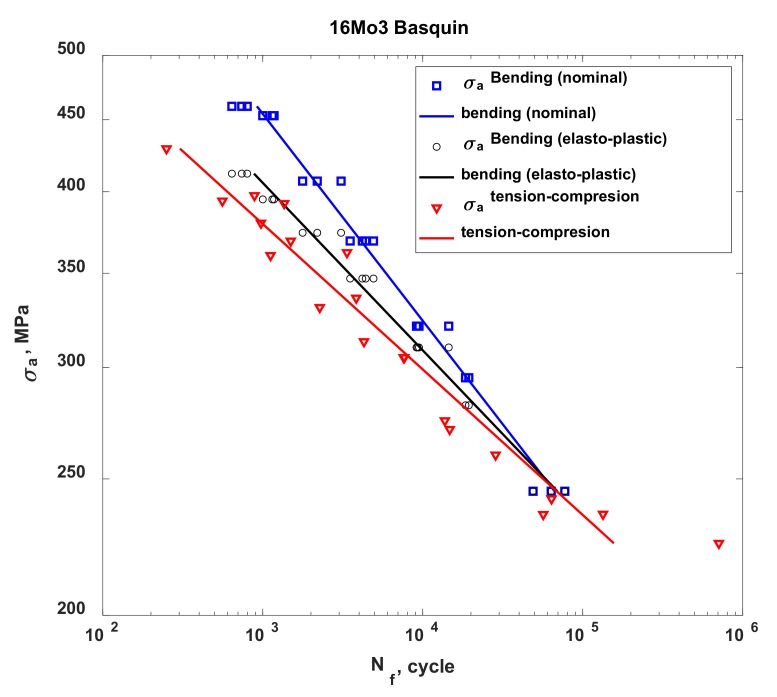
The stress characteristic for the 16Mo3 steel, developed for the cases of tension-compression and oscillatory bending.

**Figure 7:**
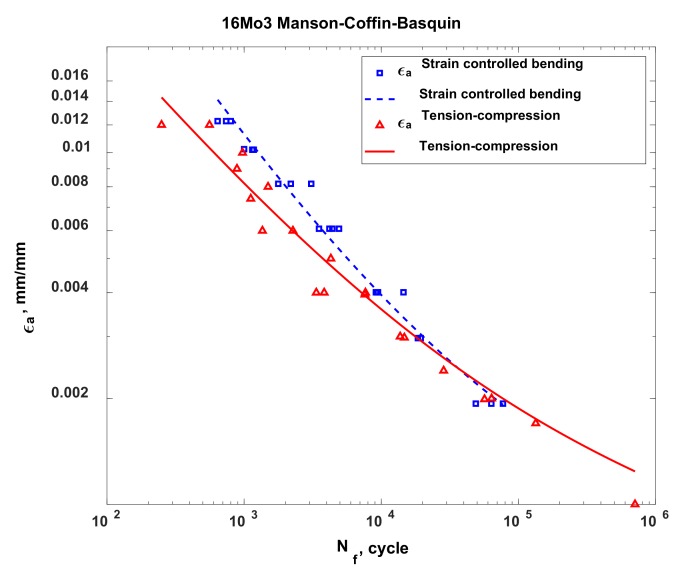
The strain characteristic for the 16Mo3 steel, developed for the cases of tension-compression and oscillatory bending.

**Figure 8:**
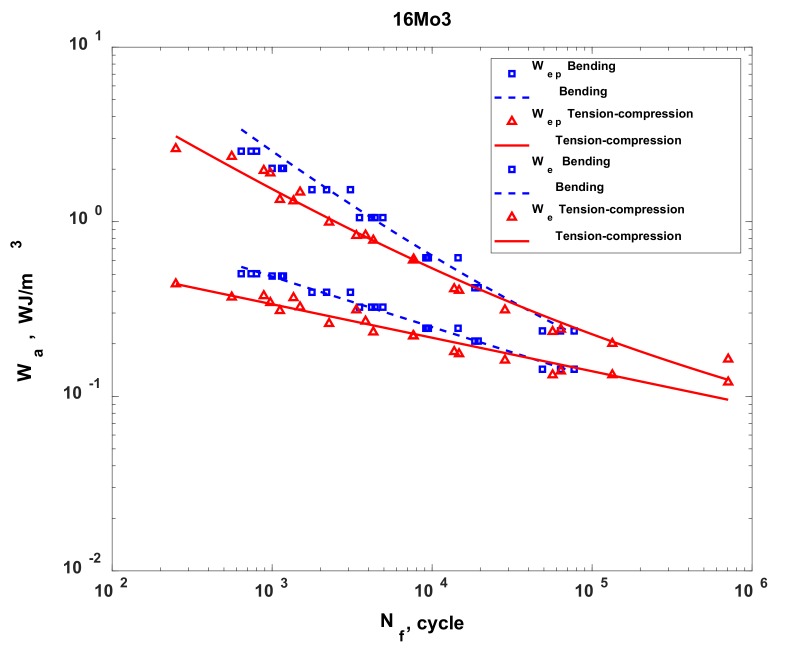
The energy characteristic for the 16Mo3 steel, developed for the cases of tension-compression and oscillatory bending.

In the case of 16Mo3 steel, for each characteristic analysed in this study (stress, strain and energy), on the basis of my own results and results taken from other studies, I determined that the fatigue life obtained under bending conditions was higher than that obtained under tension-compression conditions. This result can be explained by the considerably smaller volume of material that was subjected to maximum (destructive) loads in the case of bending, as compared to the fatigue test under tension-compression conditions. The characteristic for stress (Figure 6) indicates that the results of fatigue tests under oscillatory bending conditions performed according to the elasto-plastic model show higher strength than the fatigue tests under tension-compression conditions. However, the test results obtained according to this model are much closer to the results obtained under tension-compression conditions than to the results for rated bending. Using the elasto-plastic body model to determine the stress and strain in the test piece subjected to bending brought the obtained results much closer to those obtained from the fatigue test under tension-compression conditions. Similar to the stress characteristic, the characteristics for strain (Figure 7) and energy (Figure 8) also indicate a higher bending strength than tension-compression strength. Upon analysing these characteristics, it was observed that, in the bending test, the 16Mo3 steel was on the safe side, which suggests that the material was not sufficiently used and has a kind of buffering option. The results obtained from the fatigue tests under tension-compression and oscillatory bending conditions converged for all analysed characteristics at approximately 100,000 cycles.

**Figure 9:**
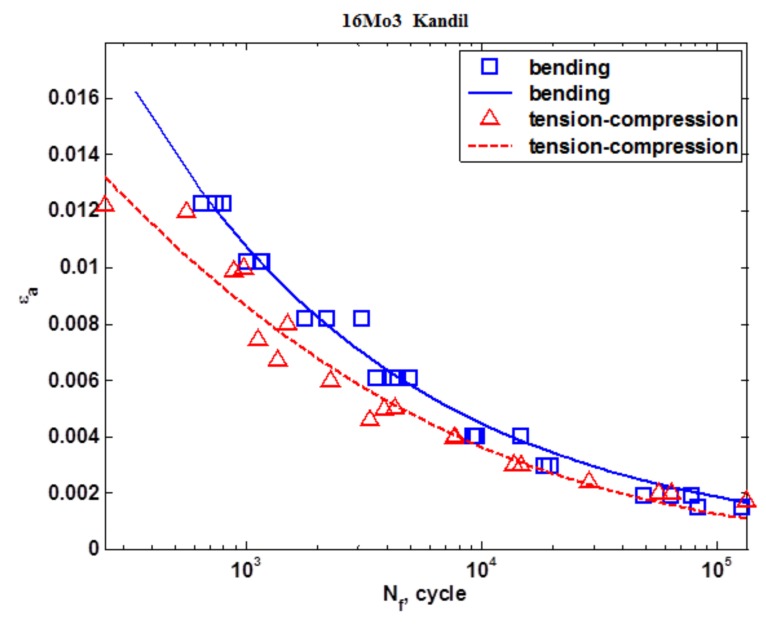
The strain characteristic according to the Kandil model.

**Figure 10:**
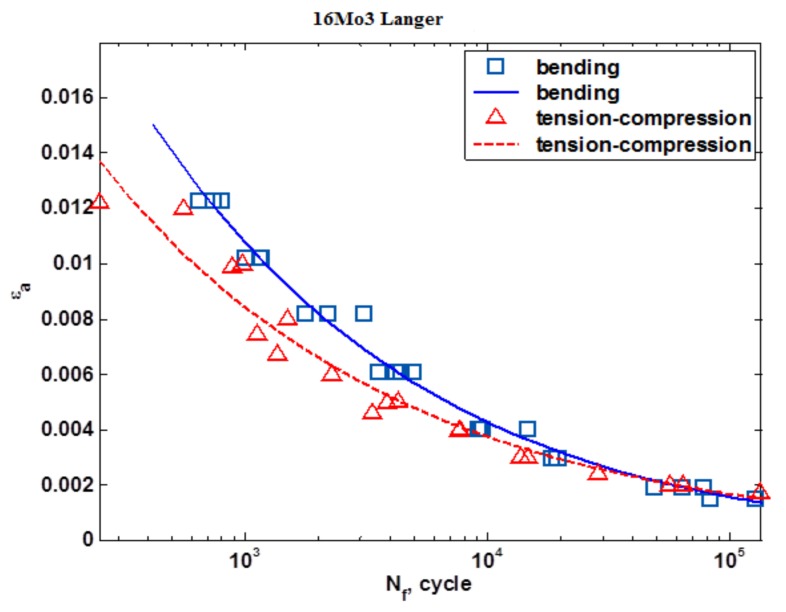
The strain characteristic according to the Langer model.

**Figure 11:**
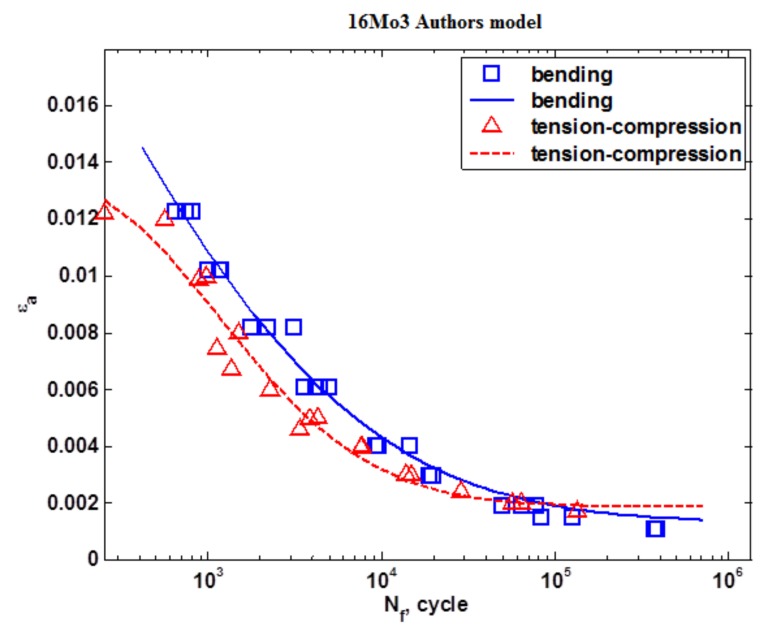
The strain characteristic according to the model proposed by the author.

Figure 9 shows the results of the fatigue tests under conditions of tension-compression and oscillatory bending that were carried out according to the Kandil model (5). Figure 10 shows the results of the fatigue tests under the conditions of tension-compression and oscillatory bending that were carried out according to the Langer model (4). Figure 11 shows the calculations that were carried out according to the model proposed by the Authors (6).

The correlation parameter $R^{2}$ was determined for all analysed curves for both cases (tension-compression and bending, Table 2). 

A data analysis showed that comparable statistical parameters were obtained for the developed models. An analysis of the fatigue curve shapes showed that, for the Langer model, the curves for bending and tension-compression are very similar to one another. The other models do not have this property. Moreover, the authors’ proposed model (6) is “S”-shaped, which is sometimes recommended for fatigue life models [21], particularly for stress specification. Both Langer and Kandil models are widely used in modern science, for example [22,23,24].

**Figure 12:**
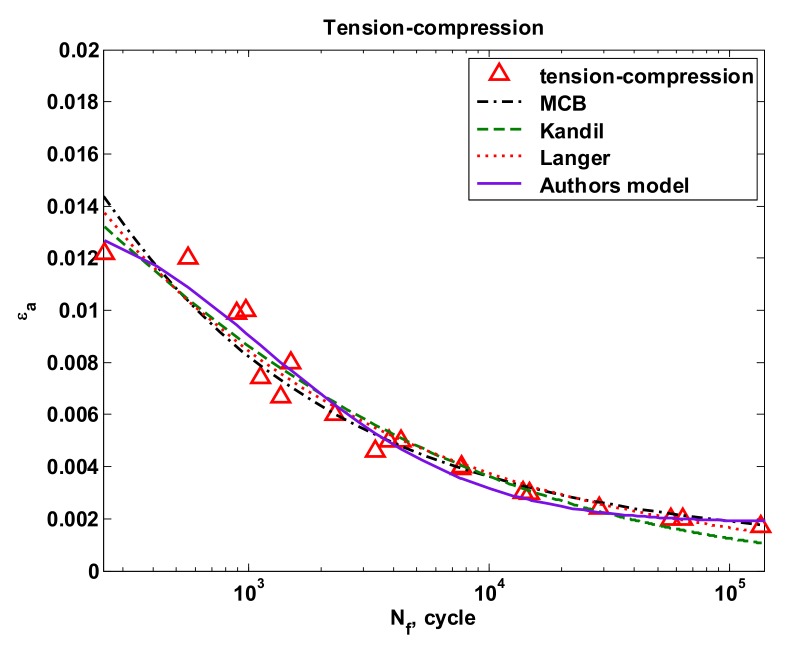
The strain amplitude for individual models under tension-compression.

**Figure 13:**
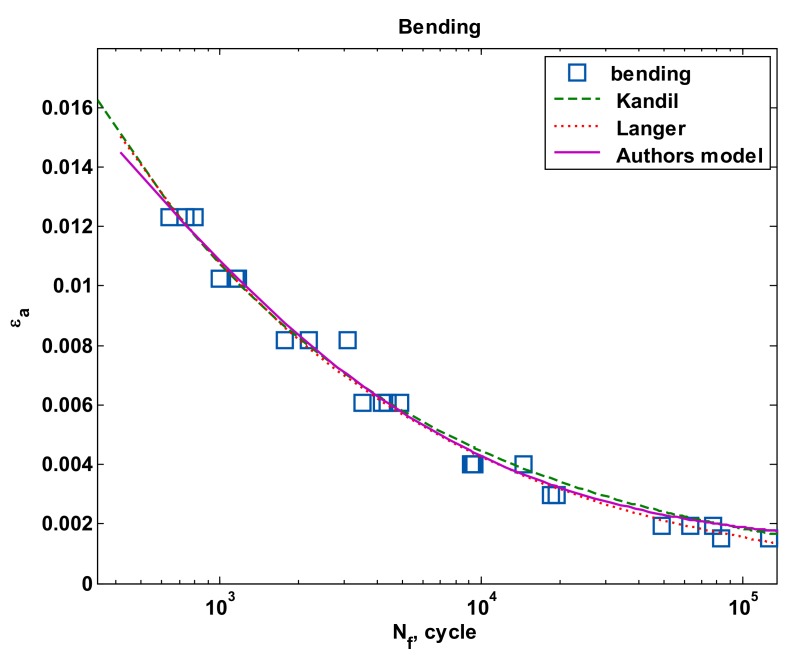
The strain amplitude for individual models under bending.

Figure 12 and Figure 13 present a comparison of curves according to all proposed models of tension-compression and oscillatory bending. For the case of tension-compression, the popular Manson-Coffin-Basquin curve (1) was added for comparison purposes.

## 4. Conclusions

- In the case of the analysed 16Mo3 steel, the characteristics in terms of stress, strain and energy determined on the basis of tests carried out under oscillatory bending conditions indicated a higher fatigue life than those determined on the basis of tests carried out under tension-compression conditions.
- In the case of the stress characteristic, the results obtained in fatigue tests carried out under oscillatory bending conditions according to the elasto-plastic model matched the fatigue life better than the results obtained in fatigue tests carried out under tension-compression conditions. However, the results of fatigue tests carried out under oscillatory bending conditions according to the elasto-plastic model were much closer to the results that were obtained in fatigue tests carried out under tension-compression conditions than to the results of fatigue tests carried out under oscillatory bending conditions according to the elastic model.
- The results of fatigue tests in terms of energy for the elasto-plastic body model show higher strength than in the case of using the elastic body model for both load types.
- According to all analysed characteristics, the results of fatigue tests for tension-compression and oscillatory bending converge at approximately 100,000 cycles, which suggests that they have the same fatigue life value for the same stress, strain and energy parameters.

The newly designed and built stand for fatigue tests allows us to control the lever’s outreach amplitude, and thus enables us to control the strain on the test piece for a low number of cycles (LCF).

## Figures and Tables

**Table 1:** Comparison of material constants for the 16Mo3 steel in fatigue tests under tension-compression and oscillatory bending conditions.

16Mo3									
Testing Conditions	Material Constants								
	E, MPa	Ramberg-Osgood		Basquin		Manson-Coffin-Basquin			
		K’, MPa	N’	A	m	σ′_f_, MPa	ε′_f_	b	C
**Bending**		-	-	21.07	6.80	-	-	-	-
**Bending (e-p)**	210,000	-	-	24.91	8.40	979.87	0.769	−0.116	−0.580
**Tension-compression**		1038	0.133	27.94	9.67	780.39	0.233	−0.096	−0.473

**Table 2:** The correlation parameter $R^{2}$ for individual models of tension-compression and bending.

Model	$R^{2}$	
	Tension-compression	Bending
Kandil	0.9488	0.9886
Langer	0.9438	0.9859
Authors	0.9676	0.9872

## Nomenclature

*E*	Young’s modulus
*K*	coefficient of cyclic strength
*n*	exponent of cyclic hardening
*N*_*f*_	number of cycles to failure
$W_{a}$	amplitude of the energy parameter
*ε*_*a,t*_, *ε*_*a,e*_, *ε*_*a,p*_	total, elastic and plastic components of the strain amplitude, respectively
*σ*′_*f*_, *b*	coefficient and exponent of the fatigue strength, respectively
